# Supplementary material for: Genomic and Transcriptional Profiling Analysis and Insights into Rhodomyrtone Yield in Rhodomyrtus tomentosa (Aiton) Hassk
Source: Plants (Basel). 2023 Sep 1;12(17):3156. doi: 10.3390/plants12173156 (PMC10490526; doi:10.3390/plants12173156)
Supplement: Supplementary file 1 [file plants-12-03156-s001.zip › plants-2556217-supplementary.pdf]

Supplementary Table S1 List of primer used for Semi-quantitative RT-PCR

| GeneID /Target gene | Primers | Nucleotide sequences |
|---|---|---|
| CL11462. Contig1_All/Ethylenere sponsive TF ERF014 (*RtERF14*) | RtERF14F2 | GTC GTC CAC GTC GGC GTG CAG GA |
| | RtERF14R | CGA CGA CGT CGT CAT CCA GGT GA |
| CL1945.Contig1_All/Zinc transporter *(RtZnT)* | RtZnTF | GAC AGT AAC GTT GCC GTG TCA GCA A |
| | RtZnTR | GCT ATA GTT TCT TGA ACG ACG ACG A |
| CL3457.Contig3_All/Laccase-15 (*Rtlaccase-15*) | Rtlaccase15F | ATG AAC TCA TCA AGA GCA CAG T |
| | Rtlaccase15R | GTC CAT CGG ACC ACG GAT ATC T |
| *18s rRNA* | 18SF | CAA AGC AAG CCT ACG CTC TG |
| | 18SR | CGC TCC ACC AAC TAA GAA CG |
